# Bilateral Retinal Vasculitis in Systemic Lupus Erythematosus: A Case Series of Three Patients

**DOI:** 10.7759/cureus.88626

**Published:** 2025-07-23

**Authors:** Mohammad Abul Kalam Azad, Akash Ahmed Alif, Nayan Kumar Mallik, Sharmin Nahar, Abul khair Ahmedullah, M Masudul Hassan

**Affiliations:** 1 Rheumatology, Bangladesh Medical University, Dhaka, BGD; 2 Rheumatology, Bangladesh Medical university, Dhaka, BGD

**Keywords:** fluorescein angiogram, fundoscopy, ocular manifestations, optical coherence tomography, retinal vasculitis, systemic lupus erythematosus

## Abstract

Systemic lupus erythematosus (SLE) is a long-term autoimmune disorder that affects multiple organ systems and presents with a broad spectrum of symptoms, ranging from mild, nonspecific complaints to severe, potentially life-threatening complications. It is commonly seen in females but may occur in males. Ocular involvement is common in SLE patients. These ophthalmic symptoms can develop during the course of the disease or may be the initial sign. Although rare, sudden vision loss due to retinal vessel involvement can be the first presentation of SLE. This case series highlights a rare manifestation of SLE bilateral retinal vasculitis as either the initial or subsequent presentation of the disease. The mean age with standard deviation of the patients in this series was 29.67±9.44​ years. Of the three patients, one was male and two were female. In one case, retinal vasculitis was the initial manifestation of SLE, while in the other two, it developed during the disease. All patients were treated with intravenous methylprednisolone (MP), followed by oral prednisolone at a dose of 1 mg/kg of body weight, in combination with monthly cyclophosphamide. Partial clinical improvement was observed in all cases. Retinal vasculitis, whether isolated or accompanied by involvement of other organs, should be considered in the differential diagnosis of visual disturbances in SLE patients. Early recognition and prompt treatment are critical to prevent irreversible retinal damage and complications

## Introduction

Systemic lupus erythematosus (SLE) is a chronic, relapsing, autoimmune multisystemic disease involving single or multiple organs, such as skin, joints, kidneys, central and peripheral nervous systems, eyes, lungs, heart, and blood cells [1]. SLE can affect men and women; it is significantly more common in women due to the presence of estrogen, particularly during their childbearing years [2]. Approximately one-third of patients with SLE experience ocular manifestations, which often serve as indicators of overall systemic disease activity and morbidity [3]. SLE can involve any part of the eye. The most common ocular manifestation is keratoconjunctivitis sicca affecting 25% to 33% of individuals with SLE, typically resulting from secondary Sjögren’s syndrome. Retinal vasculitis affects 3% to 29% of cases as the second most common ocular manifestation associated with SLE. It can range from mild, nonspecific symptoms to severe visual impairment, potentially leading to organ-threatening complications [4]. Severe lupus retinopathy with multiorgan involvement with active disease usually correlates with poor prognosis in SLE [5].

## Case presentation

Case One

A 37-year-old previously healthy man from Bangladesh presented with sudden, rapidly worsening bilateral vision loss for one week, which was progressive, painless, without redness, dryness, or photophobia. He was limited to counting fingers at two feet on his right eye, while the left eye measured 6/18. Color vision was also impaired bilaterally; however, visual fields remained intact. He was afebrile, with a blood pressure of 130/80 mmHg and a regular pulse rate of 82 beats per minute. The systemic lupus erythematosus disease activity index 2000 (SLEDAI-2K) score was 12 points and was driven by visual disturbance, low complement levels, and high deoxyribonucleic acid (DNA) binding, categorizing the disease activity as moderate. Skin biopsy from skin lesion located on the upper back showed features of subacute cutaneous lupus.

**Figure 1:**
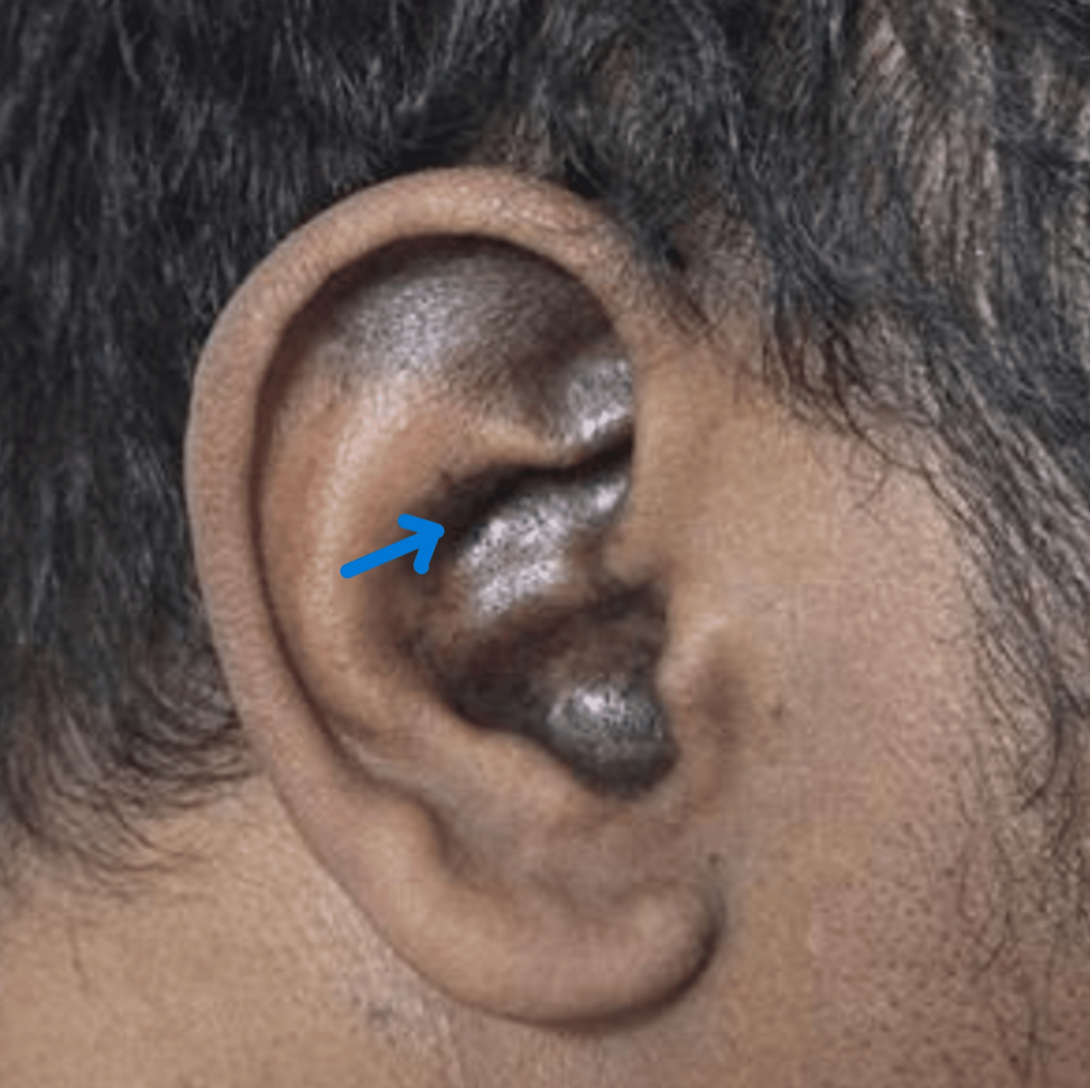
Discoid lupus in the right ear Hyperpigmented rash over the pinna of right  ears (blue arrow). Ear photograph with resolution with 600 dpi.

**Figure 2:**
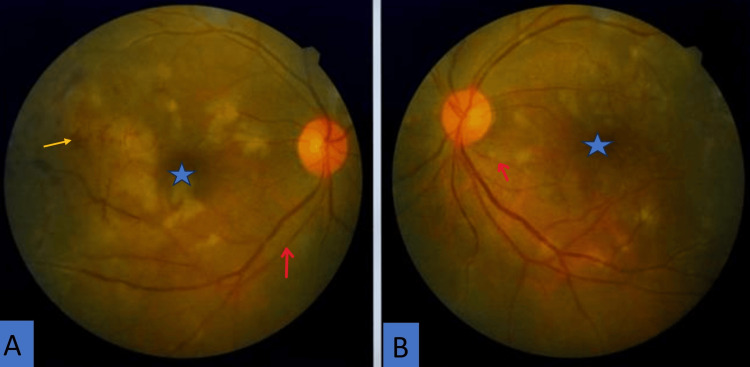
Fundal photograph of both (A) right and (B) left eye (A) right (B) left eye shows macular edema (blue star), arteriolar narrowing (red arrow), and venous segmentation, as well as a few areas of flame-shaped hemorrhages (yellow arrow). Fundal photograph with resolution of 600 dpi.

On examination, pigmentation over the right side of the pinna was seen (Figure 1). Fundoscopic examination showed bilateral cotton wool spots, more prominent in the right eye, along with arteriolar narrowing, vascular sheathing, subretinal hemorrhagic exudates, and scattered dot, blot, and flame-shaped hemorrhages (Figure 2).

**Figure 3:**
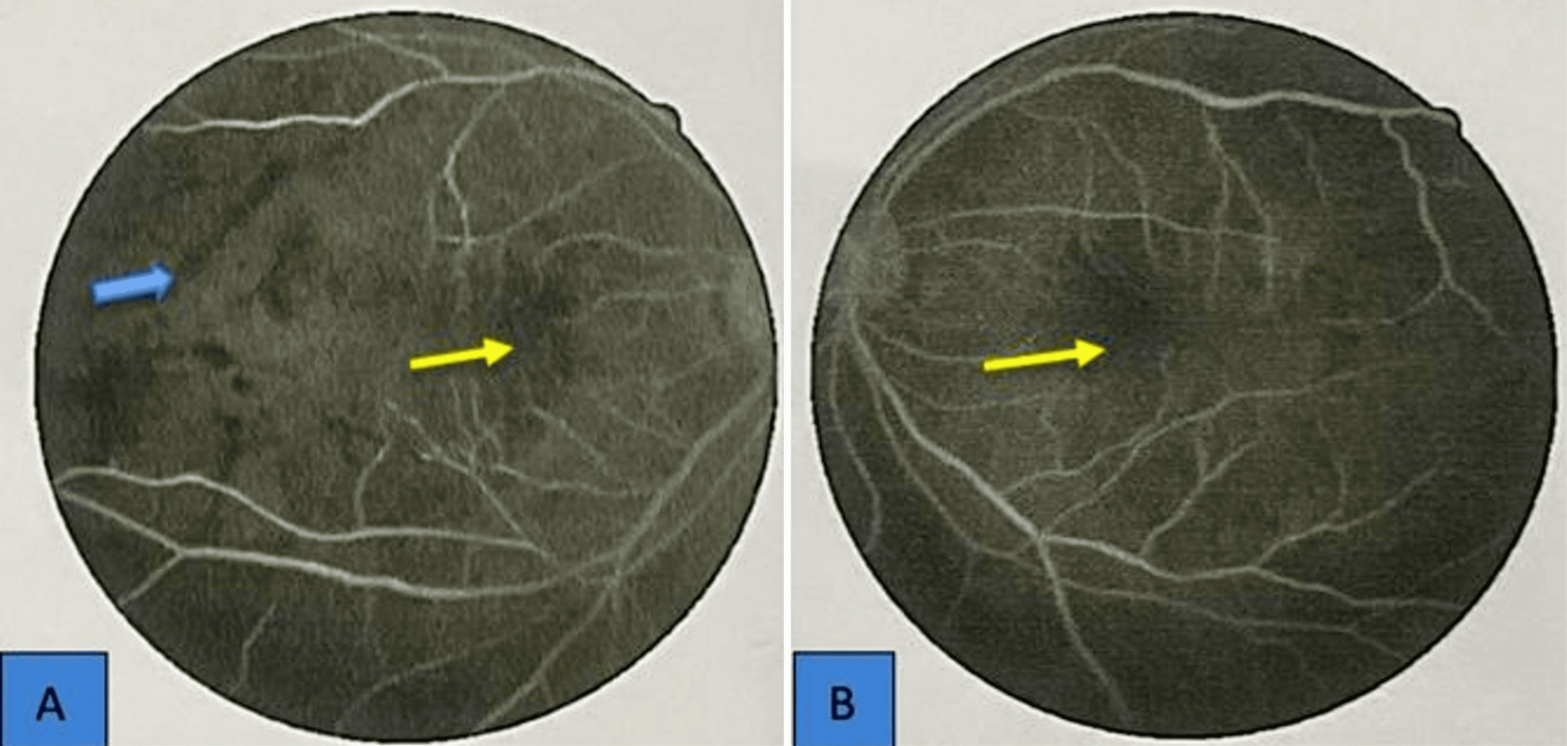
Fundus fluorescein angiography of (A) right and (B) left eye showing features of retinal vasculitis Yellow arrows show hypodense area, which is macular ischemia, and blue arrow shows retinal hemorrhage. Fundal fluorescein photograph with resolution of 600 dpi.

The neurological examination was normal. A fundus fluorescein angiogram (FFA) confirmed the presence of retinal vasculitis with occlusive retinal arteriolitis (Figure 3).

**Figure 4:**
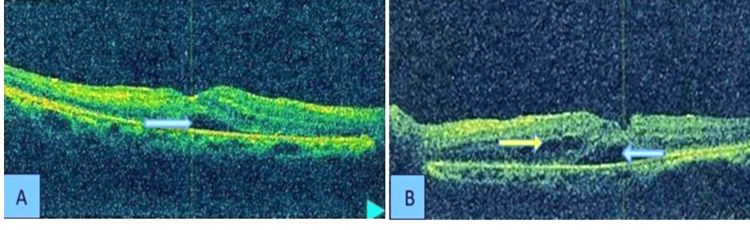
OCT image of the (A) right and (B) left eye showing features of macular edema Yellow arrow shows cystoid macular edema, and blue arrow shows subretinal fluid. Photograph of OCT with resolution of 600 dpi. OCT: optical coherence tomography.

Optical coherence tomography (OCT) showed features of macular edema, cystoid macular edema, subretinal fluid accumulation (Figure 4).

**Figure 5:**
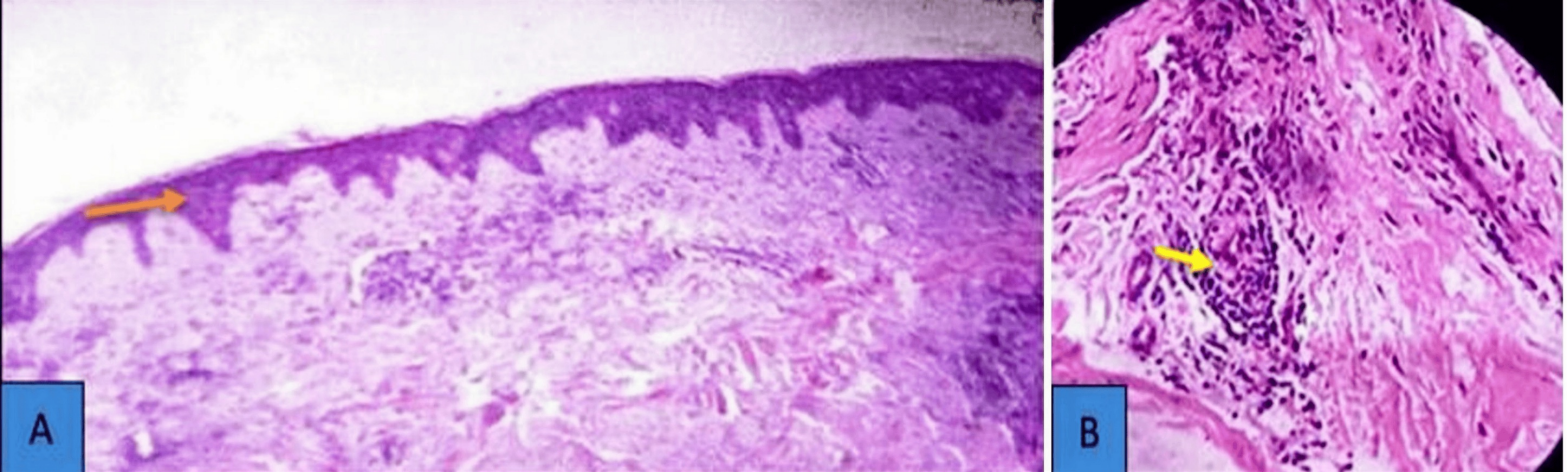
Skin biopsy showing features of subacute cutaneous lupus. Staining with hematoxylin and eosin (H&E) (A) Thickened skin with keratin buildup (orange arrow), a thin outer layer, and flattened rete pegs. (B) Lymphocyte presence around blood vessels (yellow arrow) and skin structures. Biopsy of skin photograph with resolution with 600 dpi with 1000 magnification.

Skin biopsy revealed thickened skin with keratin buildup with a thin outer layer and presence of lymphocytes around the blood vessels (Figure 5). Antinuclear antibody (ANA) was positive at a high titer on Hep-2 cells. The patient met the 2019 European Alliance of Associations for Rheumatology (EULAR)/ American College of Rheumatology (ACR) classification criteria for SLE with a score of 14. 

Case two

A woman of 33 years of age presented with sudden onset, progressive, painless dimness of vision for two weeks, more diminished on the left side, with vision reduced to counting fingers and not associated with any redness or any trauma. She also complained of a skin rash in different parts of the body for two years. The rash was insidious in onset, initially erythematous, elevated without any itching or pain, not associated with photosensitivity, and appeared during her pregnancy. Fifteen days before presentation, she experienced two episodes of generalized tonic-clonic seizures, each lasting two to three minutes, followed by unconsciousness, tongue biting, spontaneous urination, and 30 minutes of postictal confusion.

On examination, erythematous, crusted rashes of varying sizes and shapes were noted on the palms, soles, elbows, knees, and ankles. There were no clubbing or peripheral signs of infective endocarditis. No active joint inflammation or deformities were observed. Her vital sign, including BP 130/80 mm Hg, is normal. The SLEDAI-2K score of 18 points, driven by visual disturbance, low complement levels, and high DNA binding, categorized the disease activity as severe flare

**Figure 6:**
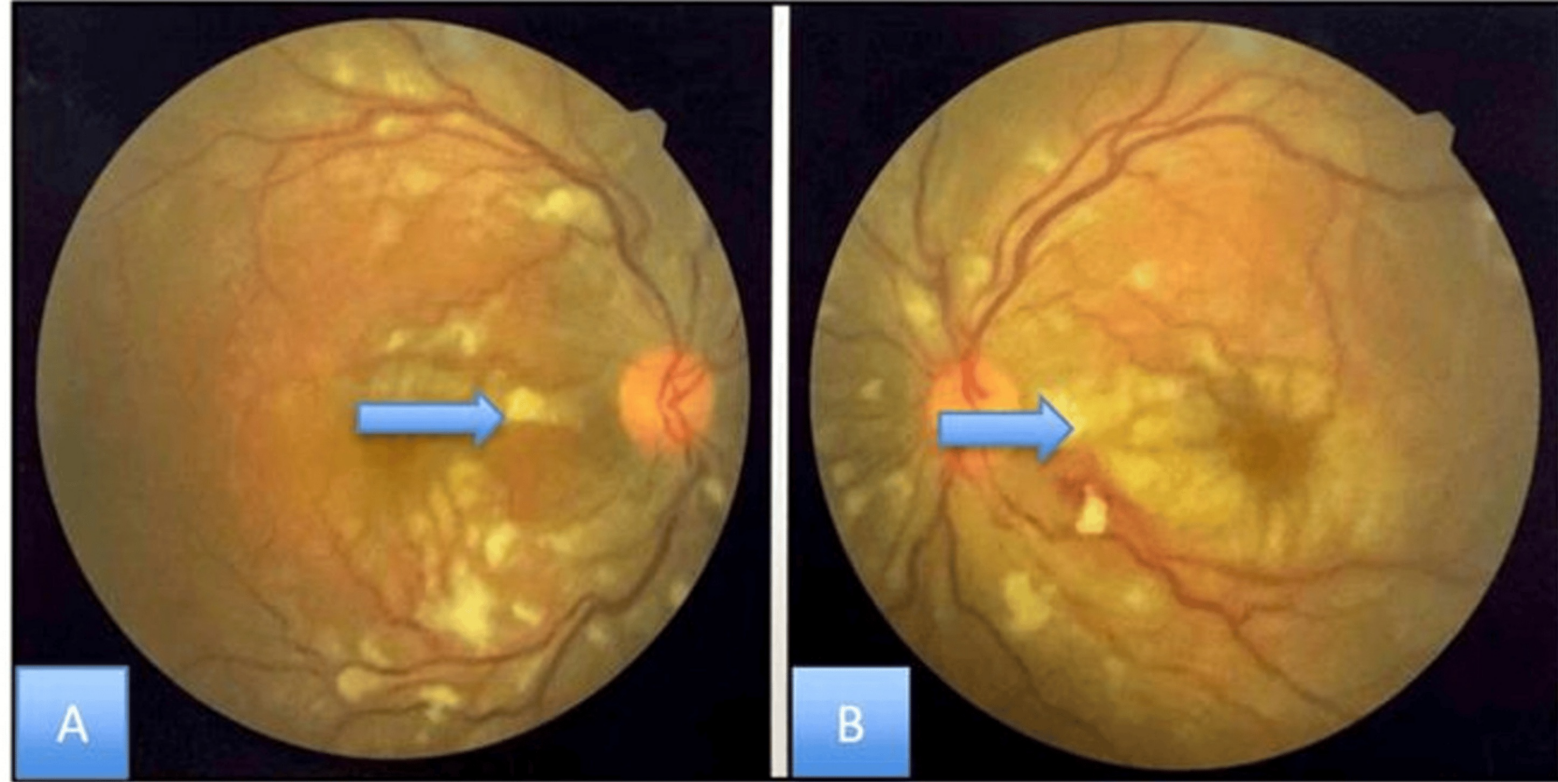
Fundal photograph of (A) right and (B) left eye shows features of retinal vasculitis Blue arrows show multiple cotton wool exudates in both eyes. Fundal photograph with resolution of 600 dpi.

Eye examination revealed a best corrected visual acuity (BCVA) of 6/36 in the right eye and counting fingers on the left eye. Fundoscopic examination revealed bilateral cotton wool spots (Figure 6).

**Figure 7:**
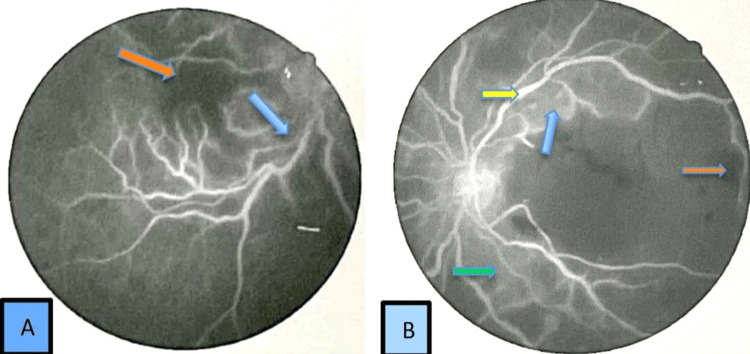
Fundal fluorescein angiogram of (A) right and (B) left eye showing features of retinal vasculitis Blue arrow indicating increased vascular leakage, yellow arrow showing vascular staining, orange arrow indicates retinal ischemia, green arrow shows perivascular sheathing. Fundal angiogram with resolution of 600 dpi.

The visual fields and color vision were impaired in the left eye. The rest of the neurological examination results were normal. A FFA revealed retinal vasculitis (Figure 7).

**Figure 8:**
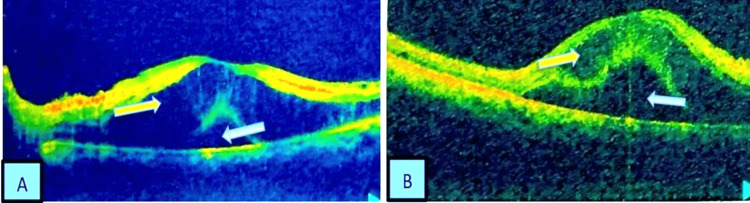
OCT image of (A) right and (B) left eye showing macular edema Yellow arrow shows cystoid macular edema and blue arrow shows subretinal fluid. Fundal OCT with resolution of 600 dpi. OCT: optical coherence tomography.

OCT image of both eyes showed cystoid macular edema and subretinal fluid (Figure 8).

ANA testing was positive at a high titer, displaying a dense fine speckled pattern on Hep-2 cells. The patient met the 2019 EULAR/ACR classification criteria for SLE, with a total score of 23 points.

Case three

A 19-year-old female from Bangladesh was admitted to Bangladesh Medical University (BMU) with a two-month history of progressive, painless dimness of vision in both eyes, without redness, dryness, photophobia, headache, or double vision. She also reported intermittent low-grade fever for six months, accompanied by erythematous, painless, non-itchy rashes on the malar area and both ear pinnae, joint pain in small joints of hands and feet, and oral ulcers. Based on these symptoms and a positive ANA test, she was diagnosed with SLE by a dermatologist and started on hydroxychloroquine 200 mg daily and prednisolone 20 mg daily. But for the last two months, she developed eye symptoms. Later symptoms became worse with features of relative afferent pupillary defect (RAPD) seen on the left eye, and visual field was impaired on the left eye.

She was prescribed prednisolone 60 mg oral. But as her condition didn’t improve, she was referred to the rheumatology department. On examination, pigmented patches were noted over the malar area and ears without erythema. No clubbing or peripheral signs of infective endocarditis were present, and no active joint inflammation or deformities were observed. Her vital signs, including BP-110/70 mm Hg, were normal and had a SLEDAI-2K score of eight points, driven by visual disturbance, and categorized the disease activity as mild to moderate flare.

**Figure 9:**
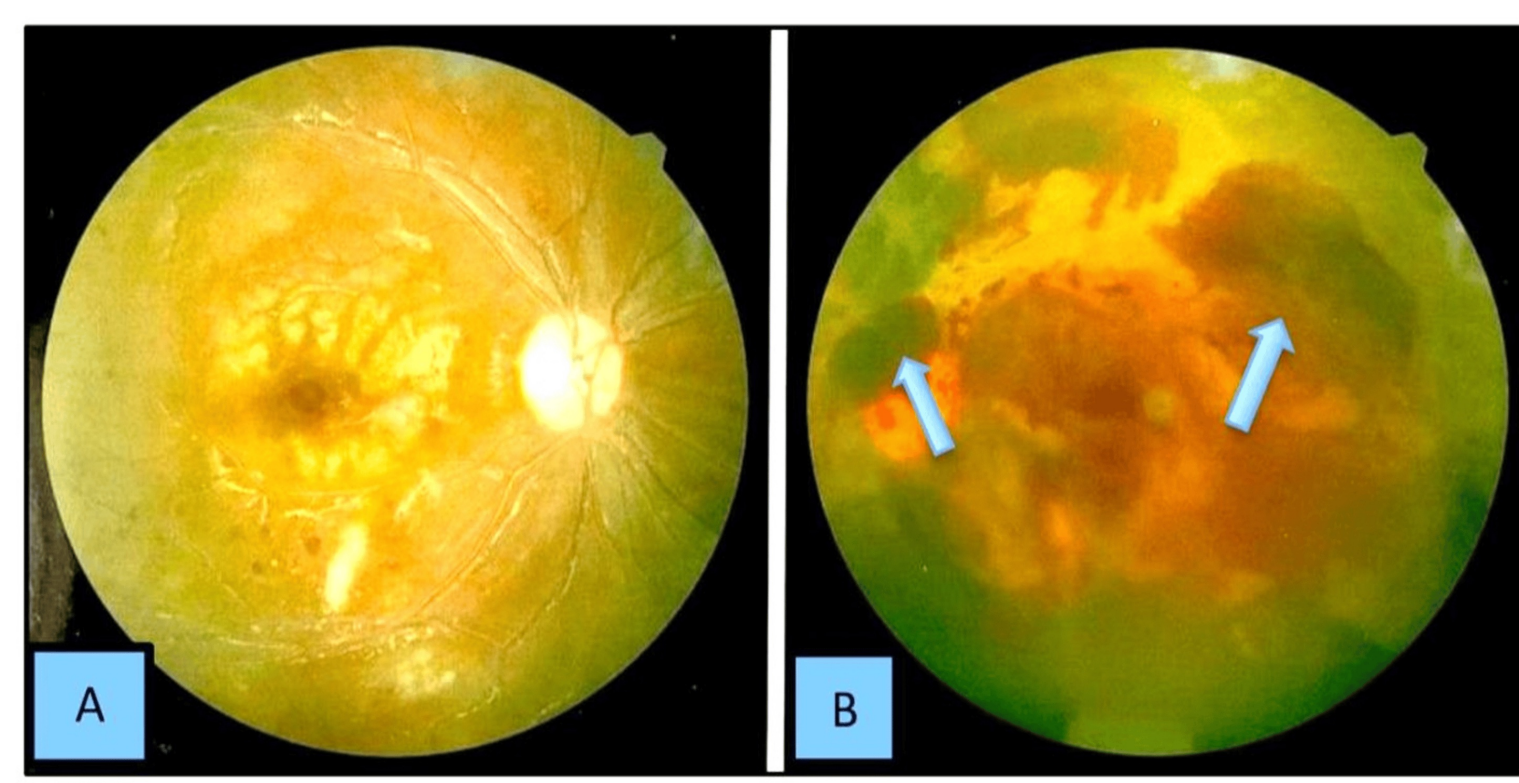
Fundoscopic examination Fundal photograph of (A) right and (B) left eye showing bilateral multiple cotton wool spots or soft exudates and; profuse vitreal and pre-retinal hemorrhage on the left eye indicated by blue arrows.

Eye examination revealed a best corrected visual acuity (BCVA) of 6/60 in the right eye and counting fingers at one foot on the left eye. Fundoscopic examination revealed active vasculitis with bilateral multiple cotton wool spots or soft exudates, and hemorrhage; profuse vitreal and pre-retinal hemorrhage on the left eye (Figure 9).

**Figure 10:**
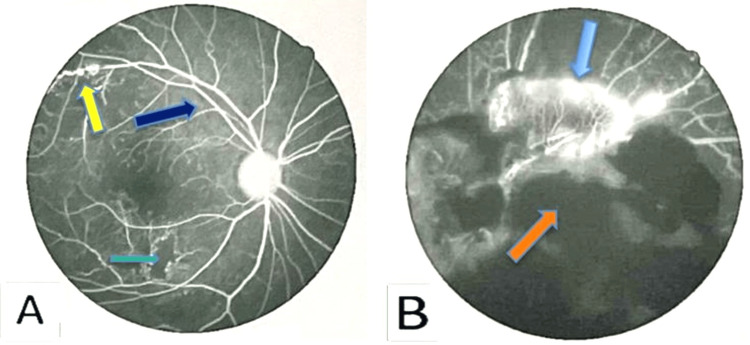
Fundal Fluorescein angiogram (A) right (B) left eye showing features of retinal vasculitis Yellow arrow indicates vascular tortuosity and bidding, green arrow shows retinal ischemia, dark blue arrow indicates vascular sheathing, blue arrow indicates increased vascular leakage, and orange arrow shows vitreal hemorrhage. Fundal angiogram with resolution of 600 dpi.

The visual fields and color vision were impaired on the left eye. The rest of the neurological examination results were normal. A FFA revealed retinal vasculitis (Figure 10).

**Figure 11:**
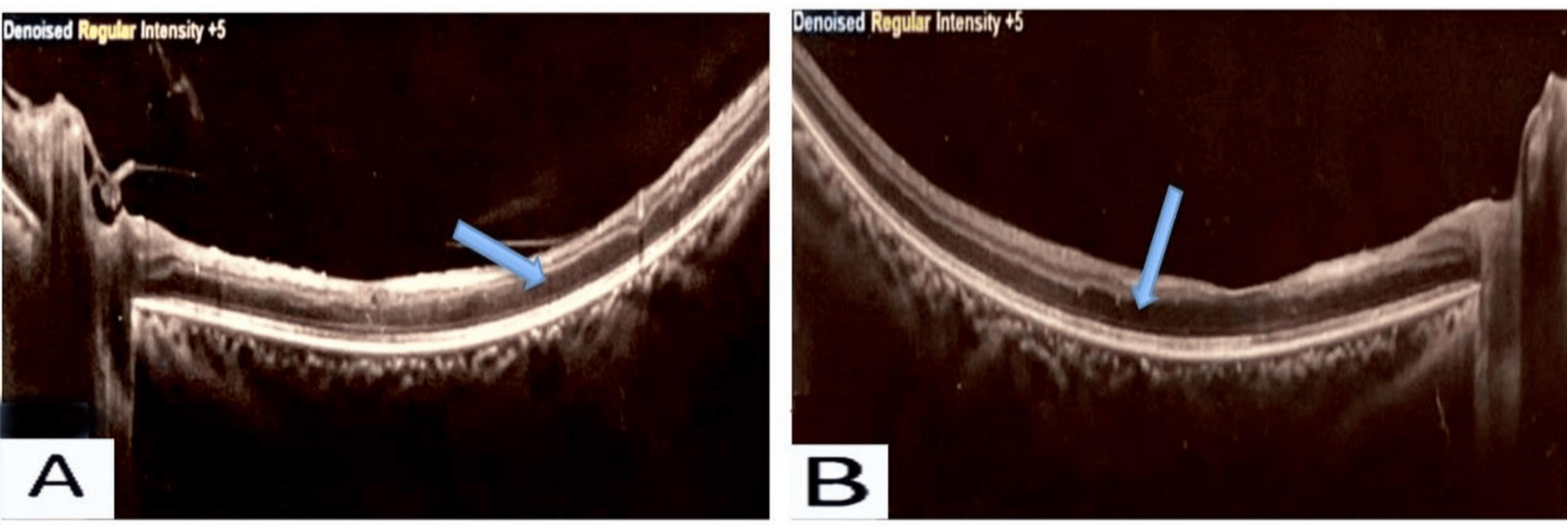
OCT image of (A) right and (B) left eye Shows features of retinal vasculitis with increased retinal thickness with areas of reduced reflectivity due to intraretinal fluid accumulation indicated by blue arrow. Fundal OCT with resolution of 600 dpi. OCT: optical coherence tomography.

The OCT image of both eyes shows an increased retinal thickness with areas of reduced reflectivity due to intraretinal fluid accumulation (Figure 11).

The patient met the 2019 EULAR/ACR classification criteria for SLE, scoring 14 points. In addition, SLEDAI-2K score was 8, primarily driven by visual disturbances. This score indicated mild to moderate disease activity.

Investigations

Blood and urine cultures were negative in cases one and three. In case two, however, the urine culture revealed Escherichia coli growth. A comprehensive diagnostic workup included screening for human immunodeficiency virus (HIV), hepatitis B and C, syphilis, and tuberculosis. Rheumatologic evaluation ruled out Behçet’s disease, anti-neutrophil cytoplasmic antibody (ANCA)-associated vasculitis, sarcoidosis, and Sjögren’s syndrome in all three cases. Rheumatoid factor (RF) was notably positive at a high titer in case two. Initial investigations for each case are shown in Table 1, and rheumatological investigation profiles are shown in Table 2.

**Table 1:** Initial laboratory investigations ACR: albumin-to-creatinine ratio, Hb: hemoglobin, TC: total count, ESR: erythrocyte sedimentation rate, CRP: c-reactive protein, RBS: random blood sugar, CXR: chest X-ray, USG: ultrasonography, SGPT: serum glutamic pyruvic transaminase, TSH: thyroid stimulating hormone, FT4: free thyroxine 4, HIV: human immunodeficiency virus, HCV: hepatitis C virus, HSV: Herpes simplex virus, TPHA: treponema pallidum hemagglutination assay, VDRL: venereal disease research laboratory, NCS: nerve conduction study, EMG: electromyography.

Name of the test	Case 1	Case 2	Case 3	Normal Range
Urine Routine examination	Pus- 0-2/HPF RBC- nil cast - nil	Protein- nil Pus-15-0/HPF	Protein- nil Pus - 0-2/HPF	All findings are absent.
Urine C/S	Negative	E. coli	Negative	No growth
ACR, mg/g	38.15	20	25	<30 g/g
Hb (Gm/dl)	10.5	9.7	10.2	M= 15+/-2 F=13.5+/- 1.5
TC of WBC/Cu mm of blood	4,800	13,000	12,000	4000-11000
Neutrophil %	73	82	85	40-75
Lymphocyte, %	20	13	13	20-40
Monocyte	6	4	02%	2-10
ESR mm in the first hour	32 mm	45mm	75 mm	0-10 (male) 0-20(female)
Blood C/S	No growth	No growth	No growth	No growth
Reticulocyte count, %	Not done	2.24	Not done	<2.5
Coombs (direct, indirect)	Not done	Negative	Not done	Negative
CRP (mg/l)	19.2	3.18	5	<8.6
RBS mmol/L	7.97	6	4.88	<11.11
HbA1C	6.8%	6.6	6.5	<5.7%
CXR	Normal	Normal	Normal	Normal
USG of the whole abdomen	Normal	Normal	Normal	Normal
S Creatinine (mg/dl)	0.76	0.63	0.64	0.7-1.3
SGPT (U/L)	51	20	24	male <45 female < 35
TSH	Not done	20.23	Not done	0.27-5.01 uIU/mL
FT4	Not done	0.80	Not done	0.71-1.76 ng/dl
Anti-Thyroid Peroxidase Ab	Not done	Positive (100.73)	Not done	0-30 unit ml
Mantoux test, mm after 72 hours	00	00	05	<10 mm
Anti-HIV (1+2)	Negative	Negative	Negative	Negative
HbsAg, Anti -HBc(total), Anti-HCV	Negative	Negative		Negative
Anti-HSV-1 IgG	Positive	Negative	Negative	Negative
Anti-HSV-2 IgG	Negative	Negative	Negative	Negative
TPHA	Negative	Negative	Negative	Negative
VDRL	Non-reactive	Non-reactive	Non-reactive	
MRI of lumbo-sacral spine	Posterior bulging at L4-L5 & L5-S1 without nerve compression	Not done	Not done	Normal finding
NCS and EMG of both lower limbs	No abnormality seen	Not done	Not done	Normal
Color duplex study of both lower limb vessels	Normal arterial system	Not done	Not done	Normal

**Table 2:** Rheumatological work-up investigations ANA: antinuclear antibody, RA: rheumatoid arthritis, ANCA: anti-neutrophil cytoplasmic antibody, Anti-CCP: anti-cyclic citrullinated peptide.

Name of the test	Case1	Case2	Case 3	Normal range
ANA	1:1280	1:320	1: 320	<1:80
Anti-ds DNA	102.09	56.4	13.72	<36
Anti smith antoibody	Not done	>400	Not done	<7 unit/ml
Serum C3	0.844	1.02	1.32	0.90-1.8 g/L
Serum C4	0.131	0.145	0.11	0.10-0.40 g/L
RA	8	128	8	<10 IU/mL
Anti-CCP	10	1.48	10	<50U/mL
c-ANCA	3.58	1.5	1.4	<5U/ml
p-ANCA	2.50	1.4	1.4	<5U/ml
Lupus Anti-coagulant	1.18	1.18	1.18	0-1.20
Anti-cardiolipin Ab Ig M and Ig G	4.6 and 5.1	4.5 and 5.2	4.5 and 5	<40 GPLU /ml
Anti-beta two glycoprotein IgM and Ig G	2.8 and 3.9	5 and5.4	3 and 5	<40 SGU U/ml

Treatment

All patients were started on pulsed intravenous methylprednisolone (1 g per day) for three days, followed by oral prednisolone (1 mg/kg/day) with a tapering regimen, and were simultaneously treated with hydroxychloroquine (HCQ) and i/v cyclophosphamide at the discretion of the rheumatology and ophthalmology teams. Both cases one and two were treated with IV methylprednisolone and IV cyclophosphamide and intravitreal Bevacizumab injection. Follow-up findings after one month for each of the cases are shown in Table 3.

**Table 3:** Follow-up after one month for all cases SLEDAI-2K: systemic lupus erythematosus disease activity index 2000.

Case		Case 1		Case 2		Case 3	
Visits		On presentation	1^st^ follow-up	On presentation	1^st^ follow-up	On presentation	1^st^ follow-up
Disease activity (SLEDAI-2K)		12 points	8 points	18 points	8 points	8 points	8 points
Visual acuity	Right eye	Counting fingers (3 feet)	Counting fingers (2 feet)	6/36	6/36	6/60	6/36
	Left eye	6/36	6/18	Counting fingers (2 feet)	Counting fingers (2 feet)	Counting fingers (1 foot)	Counting fingers (1 foot)
Field of vision	Right eye	Impaired	Impaired	Intact	Intact	Intact	Intact
	Left eye	Intact	Intact	Impaired	Impaired	Impaired	Impaired
Color vision	Right eye	Intact	Intact	Intact	Intact	Intact	Intact
	Left eye	Intact	Intact	Impaired	Impaired	Impaired	Impaired

## Discussion

Among the ocular manifestations of systemic lupus erythematosus (SLE), the most frequently observed are keratoconjunctivitis sicca, episcleritis, scleritis, and lupus retinopathy [6-7]. Among these, lupus retinopathy is notable for having the highest correlation with disease activity, as reflected by the SLEDAI [8]. However, despite its clinical significance, the current EULAR/ACR classification criteria do not include ocular manifestations as a distinct domain. Lupus retinopathy, reported in 3-29% of patients with SLE, is a well-established negative prognostic factor for overall survival [5]. In one of our patients, bilateral retinal vasculitis was the initial presenting manifestation of SLE-a rare and severe presentation. The spectrum of lupus retinopathy ranges from mild findings to more severe complications. Mild cases often exhibit cotton-wool spots, perivascular hard exudates, retinal hemorrhages, and increased vascular tortuosity. In moderate cases, additional findings such as focal or generalized arteriolar narrowing and dilated, tortuous veins may be seen. The most severe form of lupus retinopathy-vaso-occlusive retinopathy, which is characterized by occlusion of the retinal arterioles, resulting in retinal infarction or retinal vasculitis [7,9].

SLE-related retinopathy occurs through two primary pathophysiologic mechanisms. The first involves antiphospholipid antibodies inducing thrombosis in the retinal vasculature, potentially resulting in central retinal artery occlusion (CRAO) or central retinal vein occlusion (CRVO). The second pathogenic mechanism is classic lupus retinopathy, characterized by an immune complex-mediated vasculitis by releases inflammatory mediators, which initiate phagocytosis that exacerbate vascular damage [9]. Interestingly, all of our patients had normal antiphospholipid antibody levels. It has been reported by Montehermoso et al. [10] that up to 77% of SLE patients with lupus retinopathy or optic neuropathy have negative antiphospholipid serologies. This finding supports the notion that immunologic mechanisms independent of antiphospholipid antibodies can contribute to lupus-associated retinopathy. Clinically, patients with mild retinopathy may be asymptomatic, while those with severe disease often experience visual deterioration, distortion, and field defects [3]. Uniquely, vision loss was the sole presenting feature of SLE in one of our patients (case one), an atypical presentation.

Although vision loss as an initial symptom has been reported in several case studies [11-16]. Notably, most reported patients were female; only one prior case involved a male patient. In a study assessing ocular complications in SLE, 72% of eyes with lupus retinopathy demonstrated retinal neovascularization, with subsequent complications including vitreous hemorrhage in 63% of cases and retinal detachment in 27% [3]. One of our patients (case three) had vitreous haemorrhage in the left eye. The following case reports have shown the initial presentation of Retinal vasculitis in Table 4.

**Table 4:** Initial presentation of retinal vasculitis MSK: musculoskeletal.

Age/Sex	Initial Presentation	Next Presentation	Reference
32/F	Bilateral retinal vasculitis	MSK	Madurapperuma et al. [11]
34/M	Bilateral retinal vasculitis with antiphospholipid syndrome	MSK	Aldhefeery et al. [12]
14/F	Panuveitis and bilateral retinal vasculitis	Arthralgia	Alhassan et al. [13]
15/F	Purtscher-like retinopathy	Fever with myalgia and arthralgia	Palkar et al. [14]
19/F	Optic neuritis alongside retinal vasculitis	Lupus nephritis	Barkeh et al. [15]
13 and 16/F	Retinal vasculitis	Retinal vasculitis	Donnithorne et al. [16]

The most common retinal manifestation of SLE is retinal microangiopathy, which typically presents with small intraretinal hemorrhages and cotton-wool spots [17]. In these cases, visual function improved following systemic corticosteroid therapy and adjunctive intraocular Bevacizumab injections. Lupus retinopathy is associated with a higher prevalence of neuropsychiatric symptoms and autoimmune hemolytic anemia [18,19]. One of our patients (case two) had neuropsychiatric manifestations before developing eye symptoms. Retinal vasculitis in systemic lupus erythematosus (SLE) necessitates treatment directed at the underlying systemic disease. Given the severity of disease activity in our patient, high-dose intravenous methylprednisolone was administered, followed by oral glucocorticoids and cyclophosphamide. Notably, the current EULAR/ACR classification criteria for SLE do not include lupus retinopathy, although the SLE Disease Activity Index 2000 recognizes its contribution to overall disease activity.

## Conclusions

Ocular disease can be the initial manifestation of systemic lupus erythematosus (SLE) and may lead to various ocular complications. Therefore, early diagnosis and prompt management are crucial to prevent these complications. Treatment typically involves a combination of steroids and immunosuppressive drugs to control ocular manifestations. The primary goal of therapy is to achieve disease remission. Additionally, long-term follow-up is essential to monitor disease activity and manage any ongoing or emerging issues.
